# Programmed cell death associated with the formation of schizo-lysigenous aerenchyma in *Nelumbo nucifera* root

**DOI:** 10.3389/fpls.2022.968841

**Published:** 2022-09-29

**Authors:** Qinmi Xie, Hui Hou, Peixuan Yan, Haiying Zhang, Yingze Lv, Xuebin Li, Lin Chen, Danbo Pang, Yang Hu, Xilu Ni

**Affiliations:** ^1^Breeding Base for State Key Laboratory of Land Degradation and Ecological Restoration of North-Western, Yinchuan, China; ^2^Key Lab for Restoration and Reconstruction of Degraded Ecosystem in North-Western China (Ministry of Education), School of Ecology and Environment, Ningxia University, Yinchuan, China; ^3^School of Agriculture, Ningxia University, Yinchuan, China; ^4^Ningxia Helan Mountain Forest Ecosystem Research Station, State Forestry Administration, Yinchuan, China

**Keywords:** aerenchyma, programmed cell death, TUNEL, hypoxia, ethylene

## Abstract

*Nelumbo nucifera* (*N. nucifera*) is an important aquatic economic crop with high edible, medicinal, ornamental, and ecological restoration values. Aerenchyma formation in *N. nucifera* root is an adaptive trait to the aquatic environment in long-term evolution. In this study, light microscopy, electron microscopy, and molecular biology techniques were used to study the process of the aerenchyma development and cytological events in *N. nucifera* root and the dynamic changes of aerenchyma formation under the treatment of exogenous 21% oxygen, ethylene (ET), and ET synthesis i + nhibitor 1-methylcyclopropene (1-MCP). The results showed that programmed cell death (PCD) occurred during the aerenchyma formation in *N. nucifera* root. Plasmalemma invagination and vacuole membrane rupture appeared in the formation stage, followed by nuclear deformation, chromatin condensation and marginalization, and terminal deoxynucleotidyl transferase-mediated dUTP nick end labeling (TUNEL) detection was positive at this time. In the expansion stage of the aerenchyma development, cytoplasmic degradation and many vesicles appeared in the cytoplasm, and organelles began to degrade. Then the plasma membrane began to degrade, and the degradation of the cell wall was the last PCD step. After 21% oxygen was continuously filled in the rhizosphere environment of *N. nucifera* roots, the area of aerenchyma in *N. nucifera* roots was smaller than that in the control group. Moreover, ET induced the earlier occurrence of aerenchyma in *N. nucifera* root, but also, the area of aerenchyma became larger than that of the control. On the contrary, 1-MCP inhibited the occurrence of aerenchyma to some extent. Therefore, the formation of aerenchyma in *N. nucifera* root resulted from PCD, and its formation mode was schizo-lysigenous. A hypoxic environment could induce aerenchyma formation in plants. ET signal was involved in aerenchyma formation in *N. nucifera* root and had a positive regulatory effect. This study provides relevant data on the formation mechanism of plant aerenchyma and the cytological basis for exploring the regulation mechanism of plant aerenchyma formation.

## Introduction

Insufficient oxygen supply is the main cause of plant damage and death in flooded and humid environments (Voesenek et al., 2004; Rich et al., 2013). Wetland plants commonly form aerenchyma, allowing gas diffusion between roots and shoots, effectively alleviating the hypoxia damage caused by the water environment, and maintaining the normal physiological metabolism (Fan and Zhang, 2002). Therefore, wetland plants have great theoretical and practical significance for studying the development and regulation of aerenchyma.

Aerenchyma is a tissue containing many air chambers or cavities. Two mechanisms of aerenchyma formation have been described; lysigeny, in which cells die to create the gas space, and schizogeny, in which development results in cell separation (Sachs, 1882). Schizogenous aerenchyma is formed by the separation of specific cortical cells and the expansion of intercellular spaces, such as in the sheath of rice (Webb and Jackson, 1986; Kludze et al., 1993). The formation of lysigenous aerenchyma depends on programmed cell death and cell degradation, which is usually induced by various environmental stresses, such as hypoxia, drought, high temperature, and salt stress (Joshi and Kumar, 2012; Díaz et al., 2018; Sou et al., 2019; Abiko and Miyasaka, 2020). The occurrence process of the two is different, yet the final goal is the same, i.e., to create space for gas storage and transportation in plants. However, several scientists have conducted detailed research on the development of aerenchyma, and found that both schizogeny and lysigeny occurred successively during the aerenchyma formation process. Hu et al. (2016) concluded that the aerenchyma is formed by schizogeny, lysigeny, or a combination or both processes. In the process of aerenchyma formation in plants, such as, *Triticum aestivum*, *Trapa pseudoincisa*, or *Cynodon dactylon*, the phenomena of cell separation and cell degradation were observed, indicating that schizogenous and lysigenous process were involved in the aerenchyma formation, known as schizo-lysigenous aerenchyma (Song et al., 2009; Ni et al., 2018; Yuan et al., 2022). In addition, depending on the formation conditions, aerenchyma can be divided into two types: inducible and constitutive. Xerophytes generally form inducible aerenchyma; this process has been observed and studied in tomato, corn, barley, and other crops (Zhang et al., 2015; Souza et al., 2017; Mignolli et al., 2020). Wetland plants tend to form constitutive aerenchyma or form aerenchyma under oxygen-rich conditions (Kong, 2009). Yet, whether further development of aerenchyma can be induced by flooding or hypoxia is not fully understood.

The programmed cell death (PCD) of lysigenous aerenchyma is an active cell death process regulated by genetic inheritance (Frank and Dat, 2006). Yet, this process is complex and still unclear. Also, the classification of plant PCD remains debatable. Fukuda (2000) classified plant PCD into three categories according to cytological characteristics: (1) apoptosis-like PCD, which is a rapid process similar to animal cell apoptosis, with the nucleus shrinking followed by chromosome agglutination, nuclear degradation, and DNA fragmentation; (2) cell senescence and death is a slow process starting with cytoplasmic reduction, followed by nuclear and vacuole degradation; and (3) PCD regulated by vacuoles: first, the large central vacuole of the cell begins to disintegrate; then the vacuole membrane and plasma membrane break, releasing hydrolytic enzymes to degrade other organelles. Furthermore, Jones (2001) proposed classifying plant PCD into three categories according to the characteristics of plant growth and development: (1) differentiated PCD, which occurs when cells differentiate and specialize so as to form structures with special functions, such as catheters; (2) disease defense PCD, which occurs when cells are subjected to biological or abiotic stress; and (3) senescence PCD, which occurs when old cell death and nutrients are transferred to new cells. Moreover, Gadjev et al. (2008) proposed classifying plant PCD into the following four types: (1) PCD during plant development required for cell-specific function or form a structure, such as ducts, tracheid, fibroblasts, and aerenchyma; (2) PCD in plant-environment interaction, which occurs when plants are subjected to biological or abiotic stress; (3) PCD is caused by allelopathy between plants; for example, *Maceaurea maculosa* secretes catechins in soil, which induces the accumulation of H_2_O_2_ in neighboring plants roots and eventually leads to PCD (Bais et al., 2003); and (4) PCD induced by extreme environments, such as strong ultraviolet radiation and high salinity, can induce cell oxidative stress and reactive oxygen species-dependent PCD.

The cytological characteristics of PCD in lysigenous aerenchyma have been a hot topic of interest among scientists. In the process of aerenchyma formation in maize root cortex induced by ET, Wang et al. (2000) used TUNEL cell apoptosis detection kit to detect DNA fragmentation of aerenchyma formation for the first time and proved that the process was PCD. Moreover, Gunawardena et al. (2001) discovered that aerenchyma formation initiated by hypoxia or ET is a form of programmed cell death that shows characteristics in part resembling cytoplasmic cell death and apoptosis in animal cells; plasmalemma collapse and many small vesicles appeared in the cells exposed to ET or 3% oxygen. In addition, Gunawardena et al. (2001) labeled esterified and non-esterified pectins with antibodies and found that the cell wall began to change in the early stage of PCD, but the degradation of the cell wall could not be observed under an electron microscope until the later stage. Furthermore, Inada et al. (2002) found that aerenchyma formation was very rapid in rice root coleoptile; after the vacuole membrane ruptured, the cytoplasm became swelled and cell membrane began to degrade, followed by the degradation of cell content, plasma membrane, and cell wall. The main characteristics of PCD during catheter differentiation included protoplast shrinkage and nuclear DNA fragmentation, but chromatin condensation and cell wall degradation did not occur (Nakashima et al., 2000; Obara and Fukuda, 2004). Ni et al. (2014) found obvious PCD characteristics in the cavity formation of *Typha latifolia* leaves, such as the diffuse DNA gel electrophoresis pattern, positive TUNEL assay, and nuclear deformity until disintegration, vacuole rupture, and cell wall disintegration was observed under transmission electron microscopy. These studies suggest that the development of lysigenous aerenchyma involves cell degradation but also that the characteristics of cell degradation or PCD are different in different plants.

Programmed cell death of lysigenous aerenchyma is regulated by relevant signals from the external environment and plant endogenous (Kong et al., 2008). Studies have shown that hypoxia and other abiotic stresses can induce aerenchyma in *Dactylis glomerata*, *Lolium arundinaceum*, *Festuca arundinacea*, *Colocasia esculenta*, and other plants to reduce the negative impact of stress on plants (Abiko and Miyasaka, 2020; Smith, 2021). The membrane system of plant cells is gradually degraded under senescence or stress, and a lot of lipids are produced, which leads to the loss of membrane function, these lipids combine with osmic acid to form osmophilic substances with deep electron density (Papadakis et al., 2004; Yao et al., 2008). A previous report has indicated that osmiophilic globules related to the injury degree suffered by plants under adversity stress (Sam et al., 2003). Under water stress, the internal structure of chloroplast in eggplant leaves changed, the membrane structure was greatly damaged, and the number of osmiophilic particles increased; it is assumed that plastoglobuli may participate in the synthesis and recycling of lipophilic products and oxidative stress defense (Fu et al., 2013). Lorbiecke and Sauter (1999) found that the synthesis of ET increases in plants under hypoxia conditions. Moreover, Pan et al. (2022) found that the hypoxia signal induced the expression of *ACS* and *ACO* in oxygen-tolerant genotype cotton, resulting in the synthesis of more ET in this genotype cotton. Therefore, ET may be a key signal to regulate cell death and further lead to the formation of lysigenous aerenchyma. Studies suggest that both endogenous and exogenous ET can induce the formation of aerenchyma (Drew et al., 1981; Jackson et al., 1985; Jackson and Armstrong, 1999; Gunawardena et al., 2001). On the contrary, Drew et al. (2000) found that ET inhibitors (Ag^+^, 1-MCP) can significantly inhibit the formation of aerenchyma. However, some studies reported that the formation of aerenchyma in some plants is not dependent on ET regulation, such as in the case of *Juncus effususus* (Visser and Bögemann, 2006).

*Nelumbo nucifera* is an important aquatic economic crop with high edible, medicinal, ornamental and ecological restoration values (Bai et al., 2013; Sun, 2016), whose underground rhizomes and adventitious roots form aerenchyma in submerged environment. Moreover, *N. nucifera* root is easy to cultivate and grows fast under laboratory conditions, which is an ideal material for studying the development of aerenchyma and related signal regulation. At present, there are many studies on edible and medicinal components of *N. nucifera* (Yang et al., 2008; Mukherjee et al., 2010; Lee and Lee, 2011), but there is a lack of research on the adaptation mechanism of *N. nucifera* under hypoxic environment, especially the development of aerenchyma and its cytological essence and regulatory factors. In addition, the types of aerenchyma in *N. nucifera* root have not yet been identified. Therefore, in this study, light microscopy, electron microscopy, TUNEL detection, and gel electrophoresis were used to study the development of aerenchyma and cytological events in *N. nucifera* root and the dynamic changes of aerenchyma formation under treatment with exogenous 21% oxygen, ET, and ET synthesis inhibitor 1-methylcyclopropene (1-MCP). The development process and types of aerenchyma in *N. nucifera* root, the cytological characteristics of PCD, and the regulation of hypoxia stress and ET signal on aerenchyma in *N. nucifera* root were clarified. These data may reveal the formation mechanism of aerenchyma and provide the cytological basis for further exploring the regulation mechanism of aerenchyma formation.

## Materials and methods

### Materials and treatment

*Nelumbo nucifera* roots and cotyledons were cultured with seeds in a glass bottle containing 200 ml tap water in a constant temperature incubator after sowing; the culture temperature was 26°C, the illumination time was 12 h/days, and the illumination intensity was 1800 lx. After rooting, the experimental materials with consistent growth were selected and treated as follows: (1) control (CK): unaerated water; (2) continuously aerating the air (containing 21% oxygen) into nutrient solution by oxygen pump; (3) 4% ethephon was added into the nutrient solution; (4) the inhibitor of ET synthesis (1-methylcyclopropene, 1-MCP) + 4% ethephon was added into the nutrient solution; and (5) ET inhibitor 1-MCP was added into the nutrient solution. After 5 days of treatment, the root segment of *N. nucifera* was taken.

### Light microscopy

The *N. nucifera* root segment was placed in 2.5% glutaraldehyde (pH = 7.0) at 4°C overnight, and the samples were rinsed with 0.1 mol/L PBS buffer (pH = 7.0) for three times, 20 min each time. The samples were then fixed in 0.5% osmic acid for 3 h, rinsed three times in 0.1 mol/L PBS buffer (pH = 7.0), 20 min each time. The samples were dehydrated in gradient concentration of ethanol (30, 40, 50, 65, 80, and 90%, once each, 100% twice, 30 min each time), and finally transferred with 1,2-epoxypropane and embedded in Epon812 (Li and Li, 1978). Then ultra-thin slices were made with an ultramicrotome (Leica EMUC6) with a slice thickness of 1–2 μm, followed by toluidine blue staining, and observation and photographing using a light microscope. Statistics were conducted of the time, area and number of aerenchyma using software Moti Images Plus 2.0 statistics.

### TUNEL and DAPI assays

4′6-diamidino-2-phenylindole (DAPI) is a fluorescent dye that can strongly bind to DNA for nuclear staining. TUNEL assay can detect the cleavage of nuclear DNA in apoptosis. During apoptosis, DNA double strand breaks and many 3’-OH ends are exposed. The deoxyribonucleotide labeled by biotin or fluorescence can be connected to the 3’-OH ends under the action of deoxynucleotidyl transferase (TDT) enzyme, follow by the apoptotic cells or TUNEL-positive cells can be specifically and accurately detected by fluorescence excitation. Strong fluorescence indicated that the nucleus was being degraded, and weak fluorescence indicated that the nucleus was in the early or late stage of degradation. Whereas normal cells have almost no DNA breaks, so they are rarely stained, showing TUNEL negative. Positive control group: DNase I solution was added to degrade DNA strand before labeling reaction, and all nuclei were TUNEL positive. Negative control group: no TDT enzyme was added during labeling reaction, and all nuclei were TUNEL negative. The experimental steps are as follows.

First, the samples were fixed overnight in 4% paraformaldehyde and dehydrated by gradient ethanol (70, 85, and 90% once each, then twice in 100%), then embedded in paraffin, and finally prepared according to the scheme developed by Zhou and Liu (2011). The TUNEL and DAPI assays were performed according to manufacturer’ s *in situ* apoptosis detection kit (TaKaRa, Dalian, China) instructions. Fluorescence microscope (Leica DMLB microscope equipped with Leica DC 300F camera) was used for observation. The number of TUNEL-positive nuclei and DAPI-labeled nuclei in different samples were counted under a microscope and recorded. Each sample was replicated three times, and five microscopic fields (×40 objective lens) were observed for each replicate. The data were analyzed using SPSS Statistics ver. 19 software (IBM SPSS Statistics).

### DNA extraction and gel electrophoresis

The extraction method of total DNA was referred to the extraction method of DNase Plant Mini Kit (TaKaRa, Dalian, China). After sampling, about 60 mg tissue samples were frozen in liquid nitrogen immediately, and ground to ultra-fine texture using mortar and pestle. Follow by chemical cleavage was carried out. During the cleavage process, RNA, cell debris, protein and polysaccharide were removed, and the cleavage products were loaded into DNeasy Plant Maxi centrifugal column. In a short centrifugal process, DNA selectively binds to the silica gel membrane and the pollutants pass through. The remaining pollutants and enzyme inhibitors were washed and removed. Purified DNA is eluted with water or low salt buffer, to be used. To observe DNA fragmentation, samples were run with a 1,000 bp ladder on a 1.0% ethidium bromide agarose gel at a constant 60 V for about 1.5 h, and then photographed using a gel imaging system.

### Transmission electron microscopy

The samples were processed by the same method as the light microscope, and then sections with thickness of 80–100 nm were obtained by Leica EM UC6 ultramicrotome, and were mounted on the copper grids, stained with uranium acetate and lead citrate. A transmission electron microscope (Hitachi H-7650) was used for observation.

## Results

### The formation process of aerenchyma in *Nelumbo nucifera* root

The four stages of aerenchyma development were observed through continuous slices of the *N. nucifera* root in CK. The microstructure characteristics of each stage are shown below.

#### Stage 1: Stationary stage

**Figure 1:**
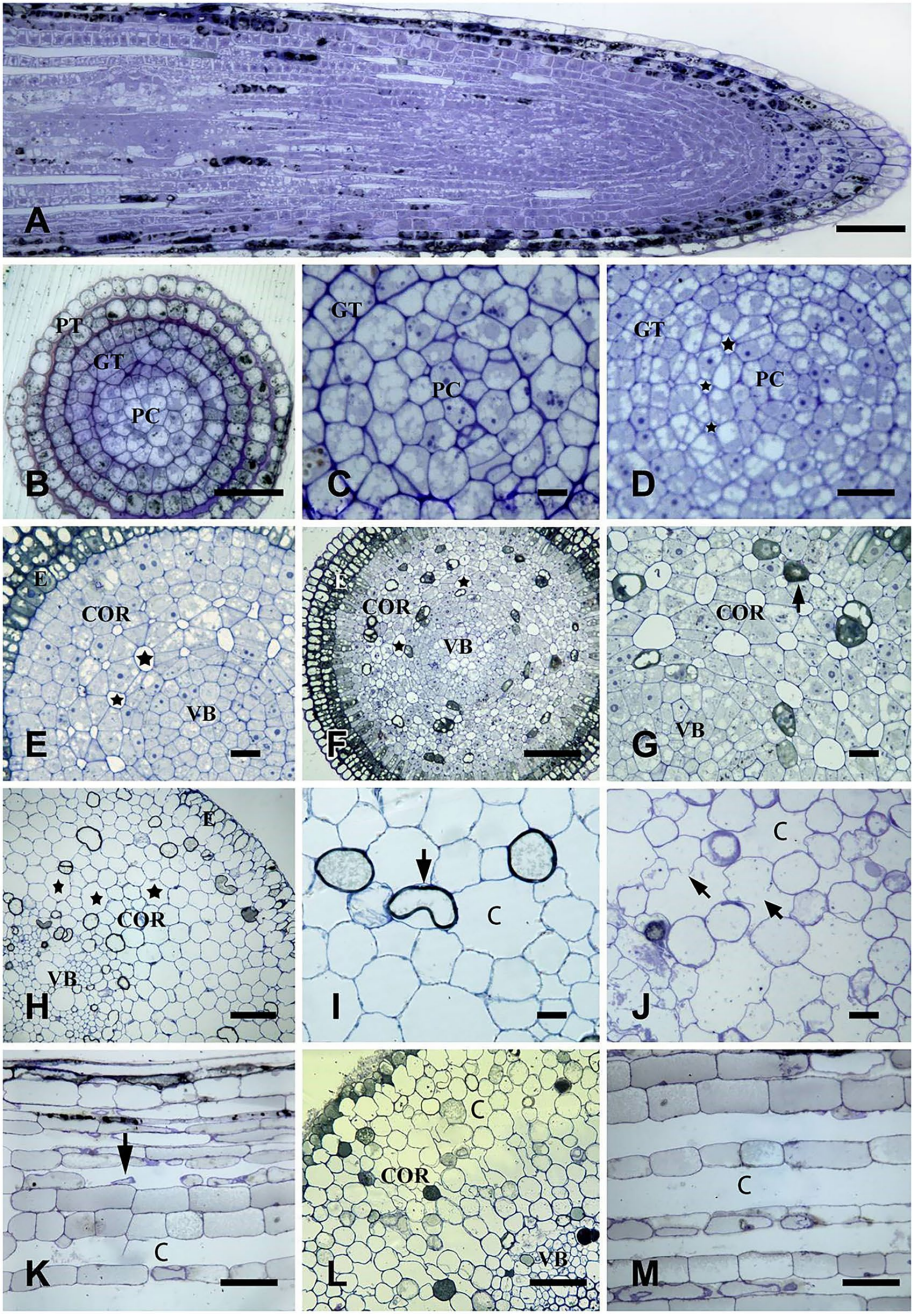
The formation process of aerenchyma in *Nelumbo nucifera* root. **(A)** 0–50 mm root tips longitudinal section and the distribution of aerenchyma. **(B,C)** The transverse section at 0.1 mm away from root tips represents a stationary stage of aerenchyma. At this stage, no intercellular space was observed. When procambium and fundamental meristem cells were enlarged, the cell morphology was regular, the cytoplasm was thick, and there were many small vacuoles in the cytoplasm. **(D–G)** Transverse sections at 0.2, 0.5, and 0.7 mm away from the root tip, represent the formation stage of the aerenchyma. **(D)** 0.2 mm: there was a small intercellular space between the cells; **(E)** 0.5 mm: the intercellular space between the cells was larger than before; **(F)** 0.7 mm: the cavities formed; **(G)** enlarged drawing of **(F)**, at this time, the cell shape becomes triangular, and the cells containing osmiophilic substances appear. **(H–K)** transverse or longitudinal sections at 20 mm and 25 mm away from the root tips, respectively, indicating the aerenchyma expansion stage. **(H)** transverse section at 20 mm from the root tips: the aerenchyma cavities increased, and the cavities are separated by only one layer of cells; **(I)** the local amplification map of **(H)** showed that there were collapsed cells around the aerenchyma cavities; **(J)** transverse section at 25 mm: cell collapsed, and the cell walls of some cells were degraded. **(K)** longitudinal section at 25 mm: there were longitudinally extended ventilation pipelines with undegraded cell debris. **(L,M)** The transverse and longitudinal sections at 40 mm from the root tip, respectively, indicate the aerenchyma Mature stage. **(L)** the transverse section at 40 mm: small cavities fusion into the radially extended larger once; **(M)** the longitudinal section at 40 mm: the cells were obviously degraded, and the diameter of the ventilation pipeline was larger than before. **(C)** cavity; COR, cortex; E, epidermis; GT, ground tissue; PT, protoderm; PC, procambium; V, vacuole; VB, vascular bundle. Bars: **A,C–E,G** = 30 μm, **F** and **H** = 80 μm, **I** = 40 μm, and others = 60 μm.

The root 0.1 mm from the root tip was in the stage of primary meristem and consisted of protoderm, ground meristem, and procambium (Figure 1B). At this stage, the cell morphology was regular, the cytoplasm was thick, the nucleus was large, there were many small vacuoles in the cell, and there was no obvious intercellular space between the cells (Figure 1C). The neat, tight, and regular arrangement between the cells was also observed from the longitudinal section, and no intercellular space was seen (Figure 1A).

#### Stage 2: Formation stage

At 0.2 mm from the root tip, cell layers of fundamental meristem obviously increased with the continuous periclinal and anticlinal division of root tip cells, and the vacuolization of cells was obvious at this stage. Also, small intercellular spaces began to appear between two layers of fundamental meristem cells close to the procambium (Figure 1D).

At 0.5 mm away from the root tip, with the further progression of the periclinal and anticlinal division of root tip cells, the layer of cells in the fundamental meristem increased, and the intercellular space increased with the separation of cells. In addition, small intercellular spaces also appeared between 3 and 5 layers of cells outward from the procambium (Figure 1E).

The root at 0.7–0.9 mm away from the root tip was the primary structure, and sieve tubes were differentiated in the primary cambium. Some cells containing osmiophilic granules were scattered in the cortex. Due to the separation between cells, each layer of cells in the cortex had obvious intercellular spaces, and the intercellular spaces near the procambium were larger than those near the protoderm (Figures 1F,G). The aerenchyma formation was also observed on the longitudinal section of the root tip (Figure 1A).

#### Stage 3: Expansion stage

At 20 mm away from the root tip, cortical cells were obviously separated, and the aerenchyma cavity was further enlarged. There were 7–9 cells around each cavity (Figure 1H), and some of these cells shrunk and became smaller, showing a tendency to degrade (Figure 1I).

At the 25 mm from the root tip, some cells around the aerenchyma cavities were contracted and deformed, and the cell walls of some cells started to degrade (Figure 1J). Network-like ventilation pipes were clearly observed on the longitudinal section of the root, and cell detachment and degradation could also be seen in the pipes (Figure 1K).

#### Stage 4: Mature stage

Aerenchyma in the root zone that has root hairs emerging has been in the mature stage. Most of the cells around the aerenchyma cavity were normal; yet there were some cells with small size and irregular shape, showing a degradation trend, and small aerenchyma cavities combined into large cavities extending radially on the transverse section (Figure 1L). In the longitudinal section, the diameter of the aerenchyma was larger than before, and a small amount of cell debris accumulated in the aerenchyma cavity (Figure 1M).

### Cleavage of nuclear DNA during aerenchyma formation in *Nelumbo nucifera* root

Nuclear DNA fragmentation in four stages of aerenchyma development was observed through continuous slices of the *N. nucifera* root in CK. Fluorescence characteristics of each stage are shown below.

**Figure 2:**
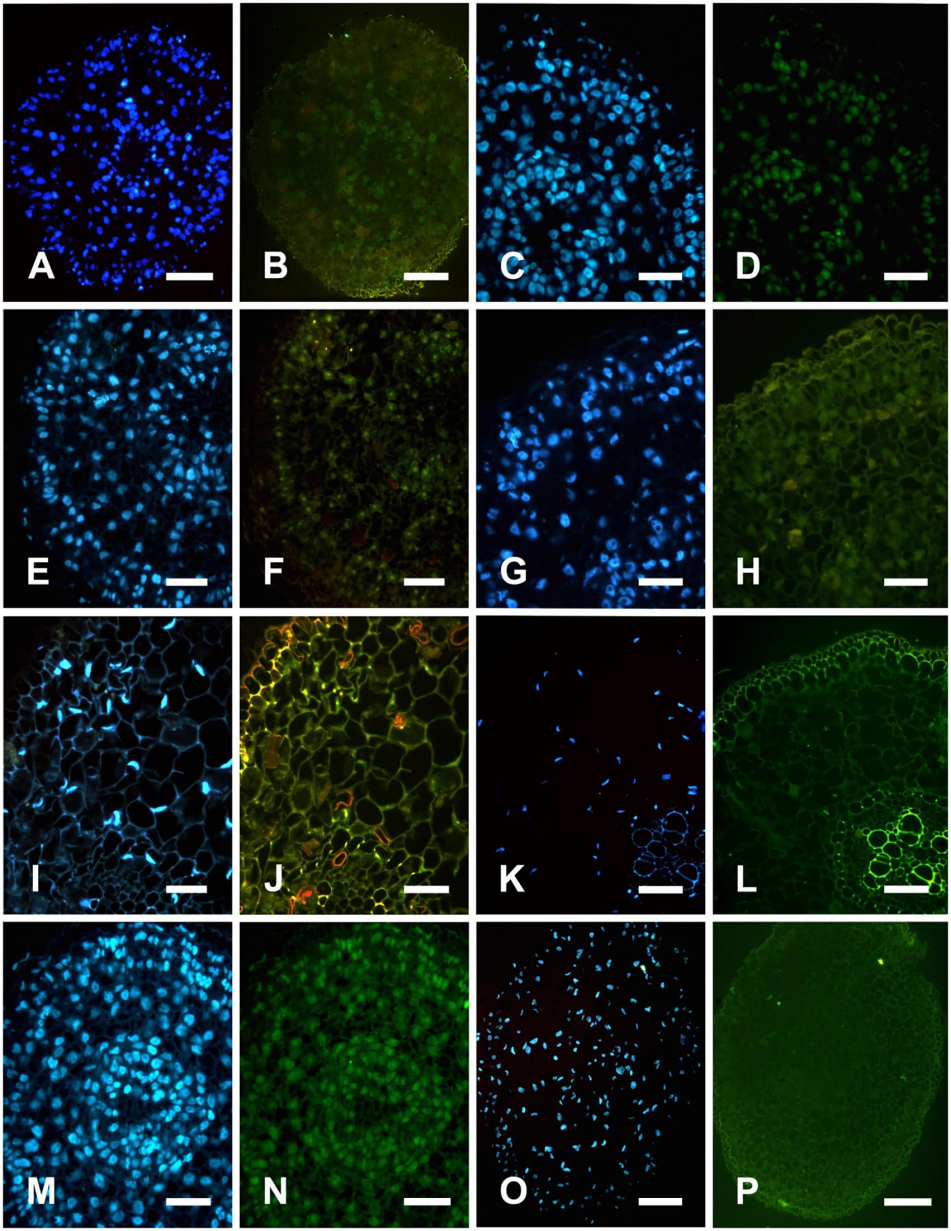
TUNEL and DAPI detect nuclear DNA fragmentation in *Nelumbo nucifera* roots. DAPI is a fluorescent dye that can strongly bind to DNA for nuclear staining. TUNEL assay can detect the cleavage of nuclear DNA During apoptosis, the apoptotic cells or TUNEL-positive cells can be specifically and accurately detected by fluorescence excitation. Strong fluorescence indicated that the nucleus was being degraded, and weak fluorescence indicated that the nucleus was in the early or late stage of degradation. Whereas normal cells have almost no DNA breaks, so they are rarely stained, showing TUNEL negative. **A,C,E,G,I,K,M,O** were DAPI staining; **B,D,F,H,J,L,N,P** were TUNEL staining. **(A,B)** In the stationary stage, most nuclei in procambium, fundamental meristem, and protoderm were stained with DAPI. Some cells in procambium showed weak TUNEL positive, indicating that the nuclear DNA of some procambium cells begin to degrade. **(C,D)** At the early formation stage, most nuclei in procambium, fundamental meristem, and protoderm were stained with DAPI. Most of the cells in the procambium and fundamental meristem were TUNEL positive, indicating that the nuclear DNA of procambium and fundamental meristem cells begin to degrade. **(E,F)** At the formation stage, most nuclei in procambium, fundamental meristem, and protoderm were stained with DAPI. Aerenchyma cavity formation and a large number of cells around the cavity were TUNEL positive and nuclear DNA was being degraded. **(G,H)** In the early expansion stage, most nuclei around the aerenchyma cavity were stained with DAPI. The number of TUNEL positive cells decreased, and the fluorescence labeling was weak, indicating that this stage was the late stage of nuclear degradation. **(I,J)** In the late expansion stage, few nuclei around the aerenchyma cavity was stained with DAPI, the nucleus was degraded into an irregular shape, and a small number of cells were TUNEL positive. **(K,L)** At the mature stage, nuclei were substantially degraded, no nucleus was stained with DAPI, and no TUNEL-positive nuclei appeared. **(M,N)** positive control: all nucleuses were stained with DAPI and marked TUNEL positive. **(O,P)** Negative control: no nucleus was stained with DAPI and no TUNEL positive nuclei were detected. Bars = 40 μm.

**Figure 3:**
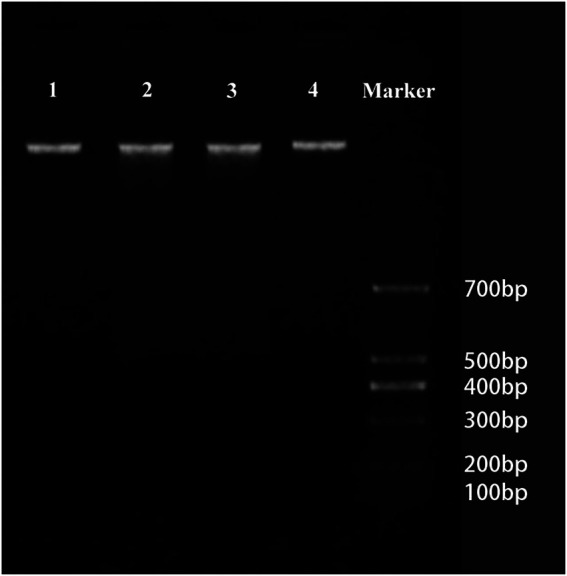
Cleavage of nuclear DNA during aerenchyma formation in *Nelumbo nucifera* by agarose gel electrophoresis. No obvious DNA smears or DNA ladders were observed in the stationary stage (lane 1), formation stage (lane 2), expansion stage (lane 3), and mature stage (lane 4), indicating that the DNA fragmentation was not detected in the DNA gel electrophoresis during the aerenchyma formation in *N. nucifera*.

In the stationary stage, the precursor cells of aerenchyma cavities showed weak yellow-green fluorescence and TUNEL-positive nuclei, whereas the nuclei of protoderm and outer fundamental meristem cells were TUNEL-negative (Figures 2A,B). In the early formation stage, most cells were TUNEL-positive and had strong fluorescence (Figures 2C,D). In the formation stage, the aerenchyma cavity was formed, and most of the nuclei around the aerenchyma cavity were TUNEL-positive, indicating that the nuclei were degraded at this stage; yet no fluorescence marker was observed in epidermal cells (Figures 2E,F). In the early expansion stage, the aerenchyma cavity further expanded with the degradation of cells, and the nucleus was TUNEL-positive, but the number of labeled nuclei decreased (Figures 2G,H). In the late expansion stage, cell degradation led to nuclear morphological abnormality, and only a small number of nuclei showed a weak fluorescence, indicating that the nucleus was in the late stage of degradation (Figures 2I,J). In the Mature stage, the nuclei were completely degraded, and no TUNEL-positive nuclei were observed (Figures 2K,L). In the positive control group, all the nuclei were TUNEL-positive (Figures 2M,N), while in the negative control group, the nuclei of the cells around aerenchyma were TUNEL-negative (Figures 2O,P). However, DNA fragmentation was not detected in DNA gel electrophoresis during the aerenchyma formation in *N. nucifera* root in CK (Figure 3).

### Cell ultrastructure of aerenchyma during PCD

Cell ultrastructure in four stages of aerenchyma development was observed through continuous slices of the *N. nucifera* root in CK. The ultrastructure characteristics of each stage are shown below.

#### Stage 1: Stationary stage

**Figure 4:**
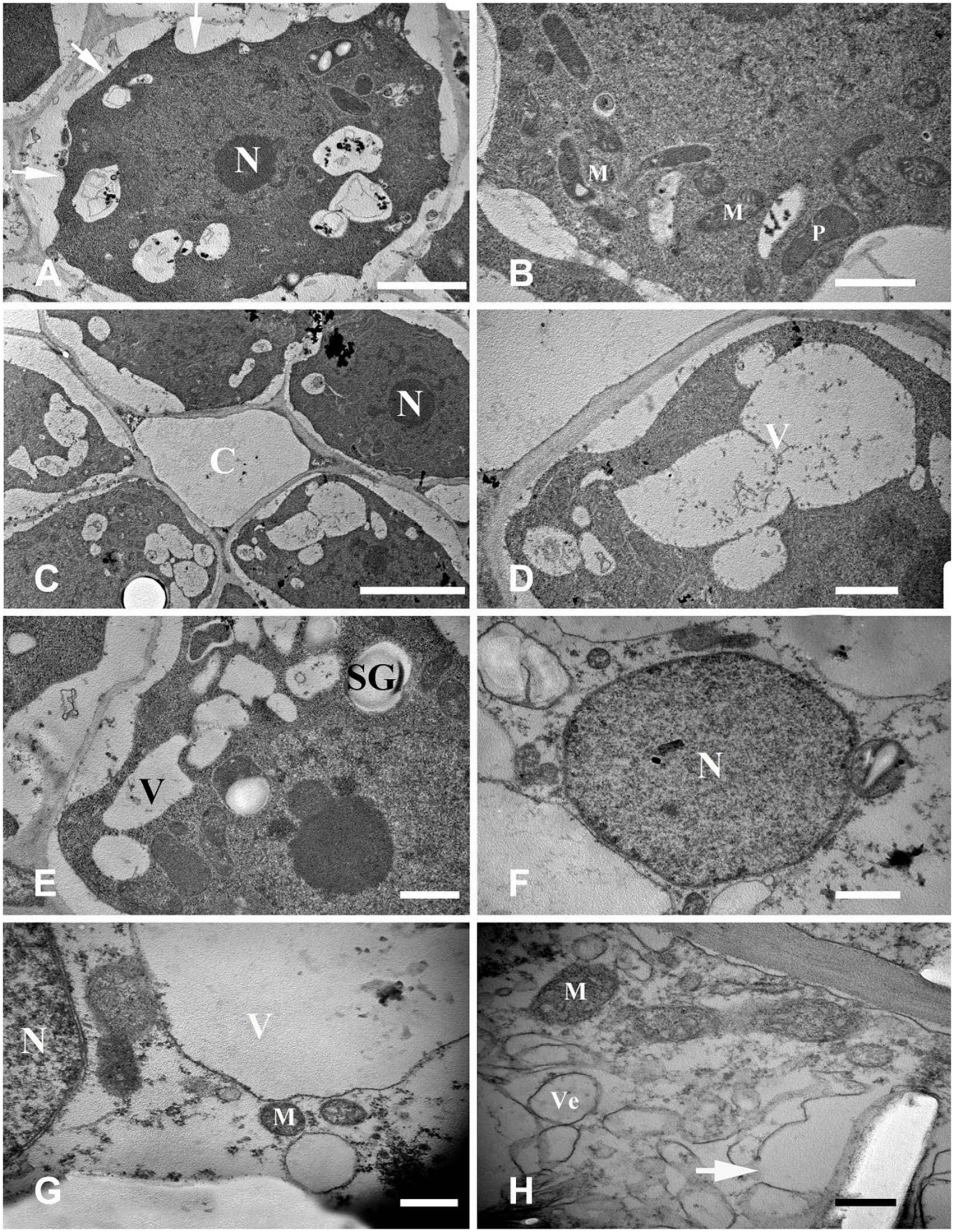
Cell ultrastructure of aerenchyma formation in *Nelumbo nucifera* roots (I). **(A,B)** The cells in the aerenchyma stationary stage have dense cytoplasm, large nucleus, multiple small vacuoles, and abundant organelles. **(C–H)** The cells in the aerenchyma formation stage. **(C)** Cavity formed between cells; **(D)** multiple small bubbles fusion into a larger one; **(E)** starch granules appear; **(F)** the “plastid ring” structure around the nucleus; **(G)** formation of the connecting strand; and **(H)** the rupture of vacuole membrane and the formation of membrane-like structure. M, mitochondria; N, nucleus; P, plastid; C, cavity; SG, starch grain; V, vacuole; and Ve, vesicle. Bars: **A** = 2 μm, **C** = 5 μm, **G,H** = 0.5 μm, and others = 1 μm.

The cells in the stationary stage had a regular shape, a large nucleus with a clear nucleolus, abundant organelles such as plastid and mitochondria, dense cytoplasm, and several small vacuoles dispersed in the cytoplasm. Also, the plasma membrane was invaginated and separated from the cell wall (Figures 4A,B).

#### Stage 2: Formation stage

**Figure 5:**
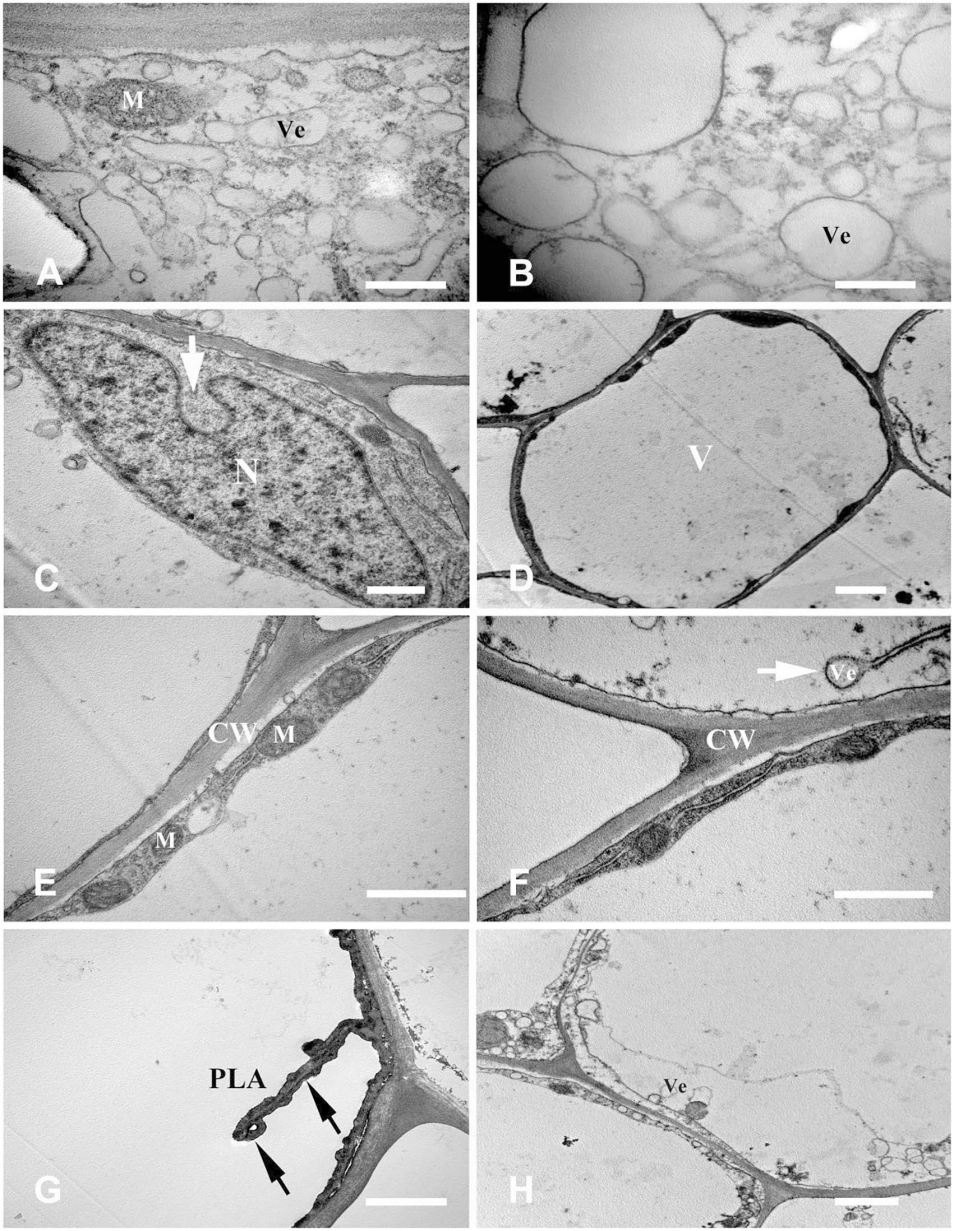
Cell ultrastructure of aerenchyma formation in *Nelumbo nucifera* roots (II). **(A–C)** The cells in the aerenchyma formation stage. **(A)** Membranous structures increased; **(B)** A small amount of granular material in the membrane structure; **(C)** Nuclear membrane invagination. **(D–H)** The cells in the aerenchyma expansion stage. **(D)** Highly vacuolar cortical parenchyma cells; **(E)** Distribution of organelles; **(F)** Endoplasmic reticulum secreted vesicle; **(G)** Plasma membrane distorted and overlapped; **(H)** Cytoplasmic degradation and vesicles appeared. M, mitochondria; N, nucleus; V, vacuole; Ve, vesicle; CW, cell wall; and PLA, plasma membrane. Bars: **A,B** = 0.5 μm, **C,E,F** = 1 μm, **D,G,H** = 2 μm.

The fundamental meristem cells near the procambium were observed with a transmission electron microscope. The number of vacuoles was higher than that observed in the stationary stage (Figure 4C), and there were a few small vacuoles fused into larger vacuoles (Figure 4D). Moreover, starch granules synthesized by plastids were also observed (Figure 4E). With cell development, most of the small vacuoles were fused into large vacuoles and occupied most of the cell volume; a clear “plastid ring” was formed around the nucleus. Mitochondria and plastids were still clearly visible (Figure 4F); the organelles were distributed near the nucleus along the connecting strand (Figure 4G). Cytoplasm concentration decreased, showing a degradation trend. The local vacuole membrane was degraded, membrane-like structures appeared (Figure 4H), and its number and volume gradually increased (Figures 5A,B). Nuclear membrane invagination and chromatin condensation are shown in Figure 5C.

#### Stage 3: Expansion stage

**Figure 6:**
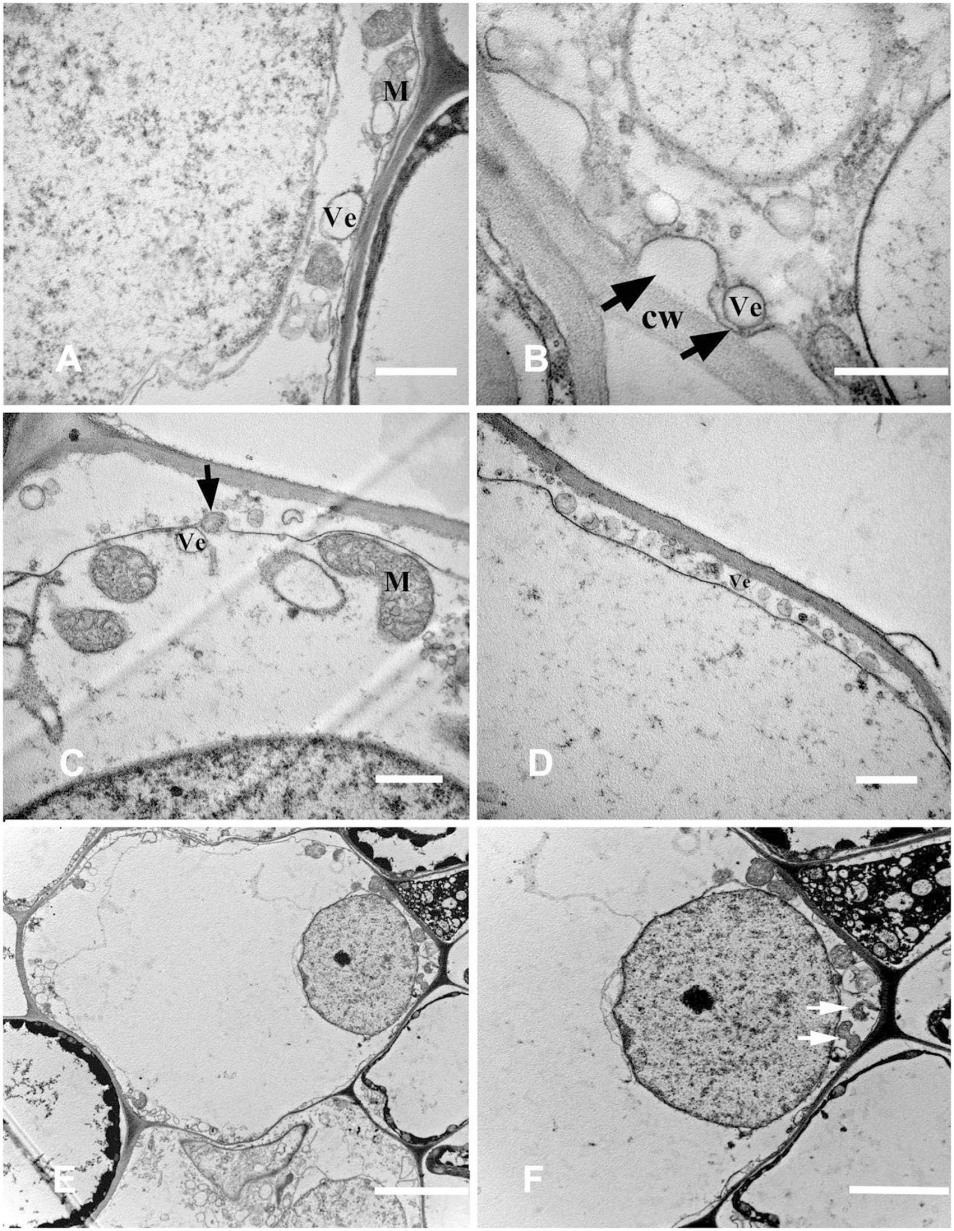
Cell ultrastructure of aerenchyma formation in *Nelumbo nucifera* roots (III). **(A–F)** The cells in the aerenchyma expansion stage. **(A)** Distribution of vesicles and distortion of organelles; **(B)** Fusion of vesicular membrane and plasma membrane; **(C)** Exocytosis; **(D)** The vesicles were transported outside the plasma membrane by exocytosis; **(E)** Complete degradation of cytoplasm and degradation of organelles; and **(F)** Undegraded organelles. M, mitochondria; Ve, vesicle; CW, cell wall. Bars: **A,E,F**  = 1 μm, **D** = 2 μm, **B** = 0.5 μm, and **C** = 5 μm.

Highly differentiated parenchyma cells were seen in the cortex, and the cytoplasmic components were squeezed at the edge of the cell by a large central vacuole, which made the cytoplasm condensed, and the electron density increased again (Figure 5D). The organelles, such as mitochondria, plastids, and endoplasmic reticulum, were still visible in the cytoplasm (Figure 5E), and the endoplasmic reticulum secreted vesicles could be observed (Figure 5F). The plasma membrane was contorted and overlapped (Figure 5G). The cytoplasm was degraded again, and many vesicles were observed (Figure 5H); the vesicles were close to the plasma membrane, and there were distorted organelles around them (Figure 6A). Subsequently, the vesicle membrane fused with the plasma membrane (Figures 6B,C), and vesicles were then transported outside the plasma membrane, and the granular substance was found in the vesicles (Figure 6D). At this time, the cytoplasm and single-layer membrane organelles were completely degraded (Figure 6E), and only a small amount of undegradable mitochondria and plastid residues were observed (Figure 6F). Nuclear membrane invagination and chromatin condensation were observed, but nucleoli were clear (Figure 6F).

#### Stage 4: Mature stage

**Figure 7:**
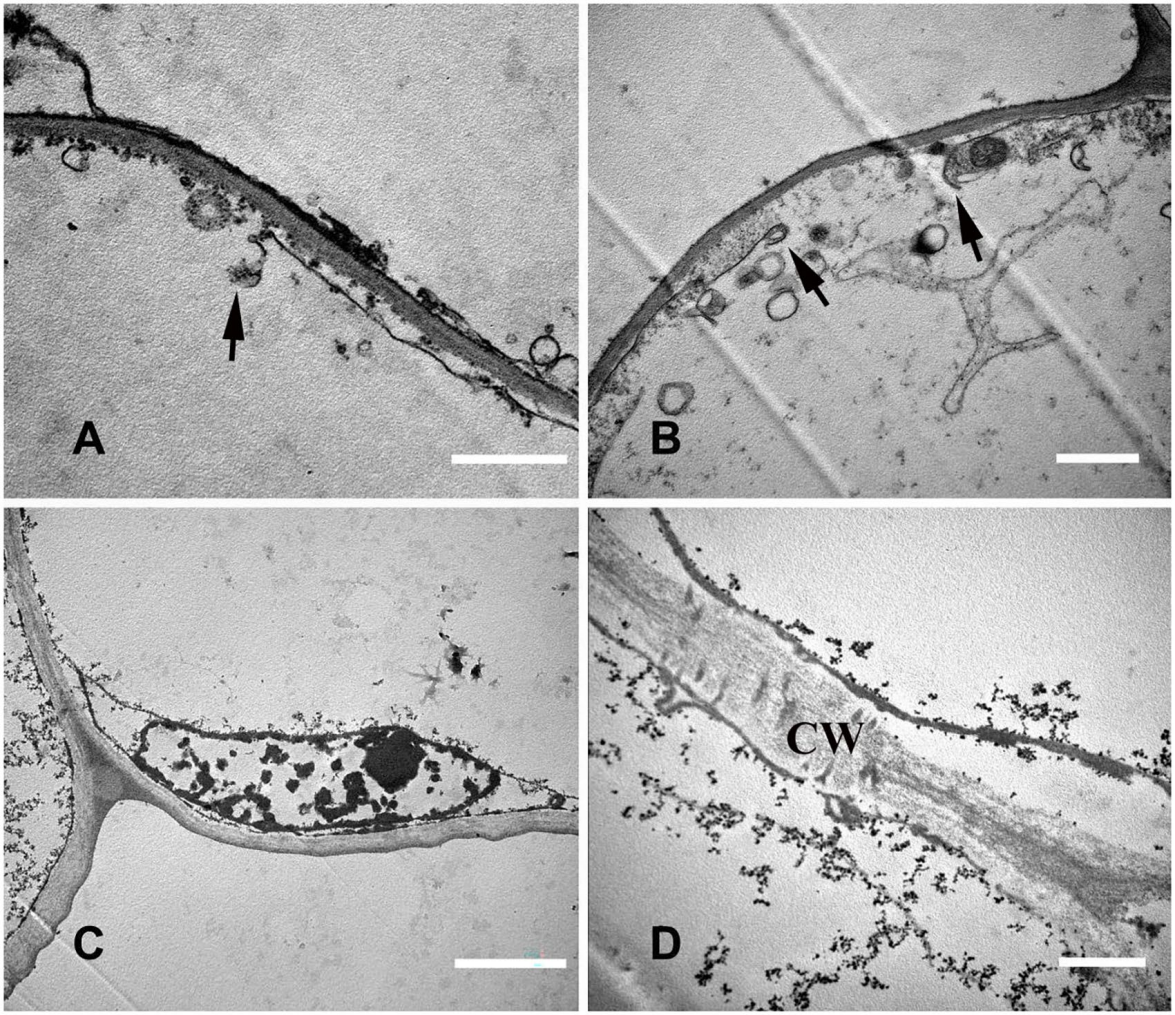
Cell ultrastructure of aerenchyma formation in *N. nucifera* roots (IV). **(A—D)** The cells in the aerenchyma Mature stage. **(A,B)** Plasma membrane degradation; **(C)** Nuclear degradation; and **(D)** Cell wall degradation. N, nucleus; CW, cell wall. Bars: **A,B** = 1 μm, **C** = 2 μm, and **D** = 0.5 μm.

The cytoplasm and organelles were all degraded at this stage, the plasma membrane was broken (Figures 7A,B), and the nuclear chromatin was highly degraded (Figure 7C). Finally, the decrease in electron density of the cell wall indicated that the cell wall began to degrade (Figure 7D).

### The effect of oxygen on the formation of aerenchyma

*Nelumbo nucifera* roots at 0.2, 0.5, 0.9, 25, and 40 mm from the root tip were selected, representing the early formation stage, middle formation stage, late formation stage, expansion stage, and mature stage of aerenchyma, respectively. With a normal water environment as the control, the development of aerenchyma after continuous aeration of 21% oxygen in the water environment was observed.

**Figure 8:**
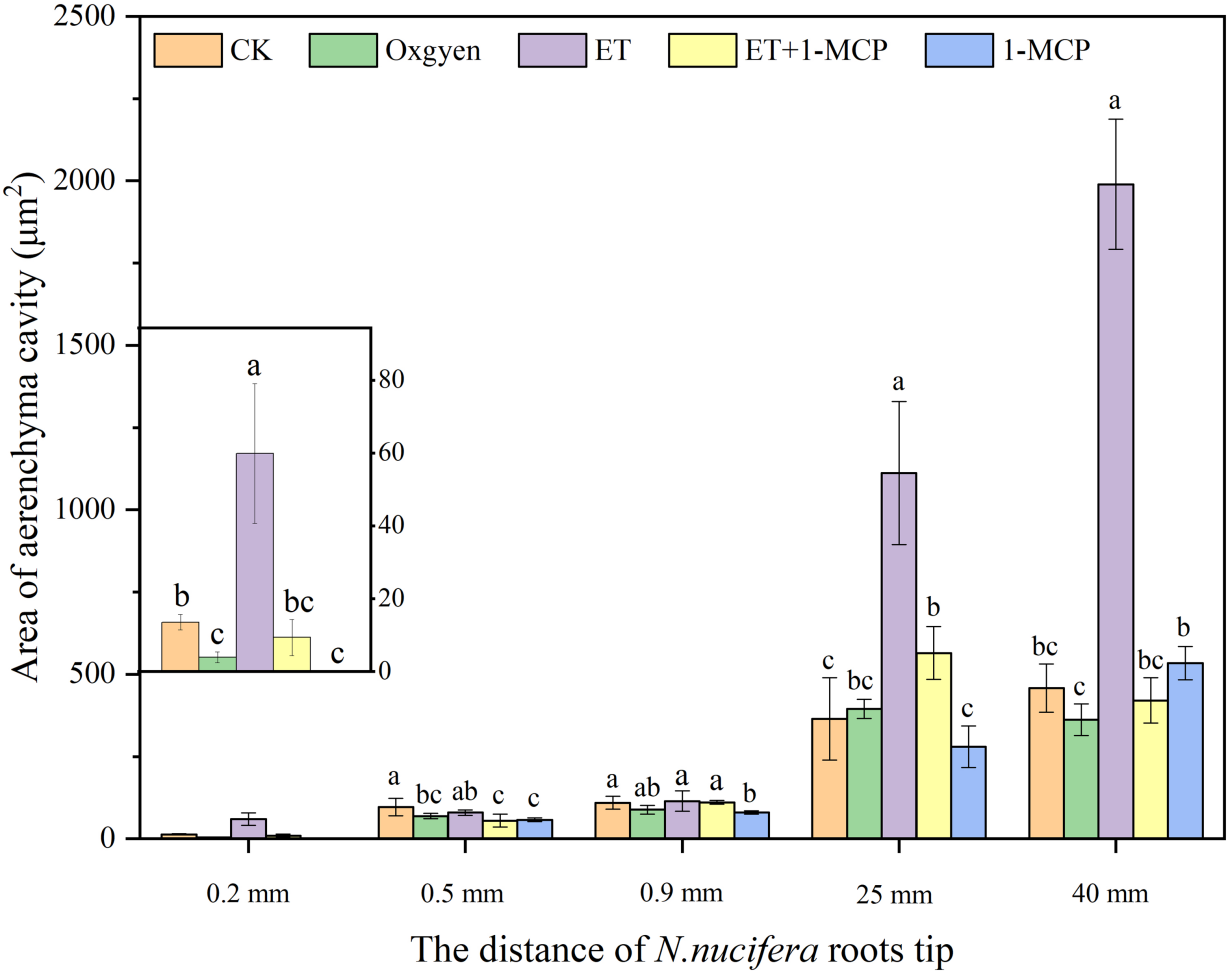
Effects of oxygen and ET on aerenchyma area in *Nelumbo nucifera* roots. *Nelumbo nucifera* roots at 0.2, 0.5, 0.9, 25, and 40 mm from the root tip were selected, representing the early formation stage, middle formation stage, late formation stage, expansion stage, and mature stage of aerenchyma, respectively. With a normal water environment as the *control, aerenchyma development after continuous aeration of 21% oxygen, adding ET and its inhibitor 1-MCP in an aquatic environment was observed, and the area of the first round of aerenchyma was counted. Values are mean ± SE (*n* = 3). Lowercase letters indicate significant differences between different treatments at the same sampling time (*p* < 0.05). Control (CK), ethylene (ET), ET synthesis inhibitor (1-methylcyclopropene, 1-MCP). CK: unaerated water; Oxygen: 21% oxygen solution; ET: ET solution; ET + 1-MCP: ET + 1-MCP solution; and 1-MCP: 1-MCP solution.

**Figure 9:**
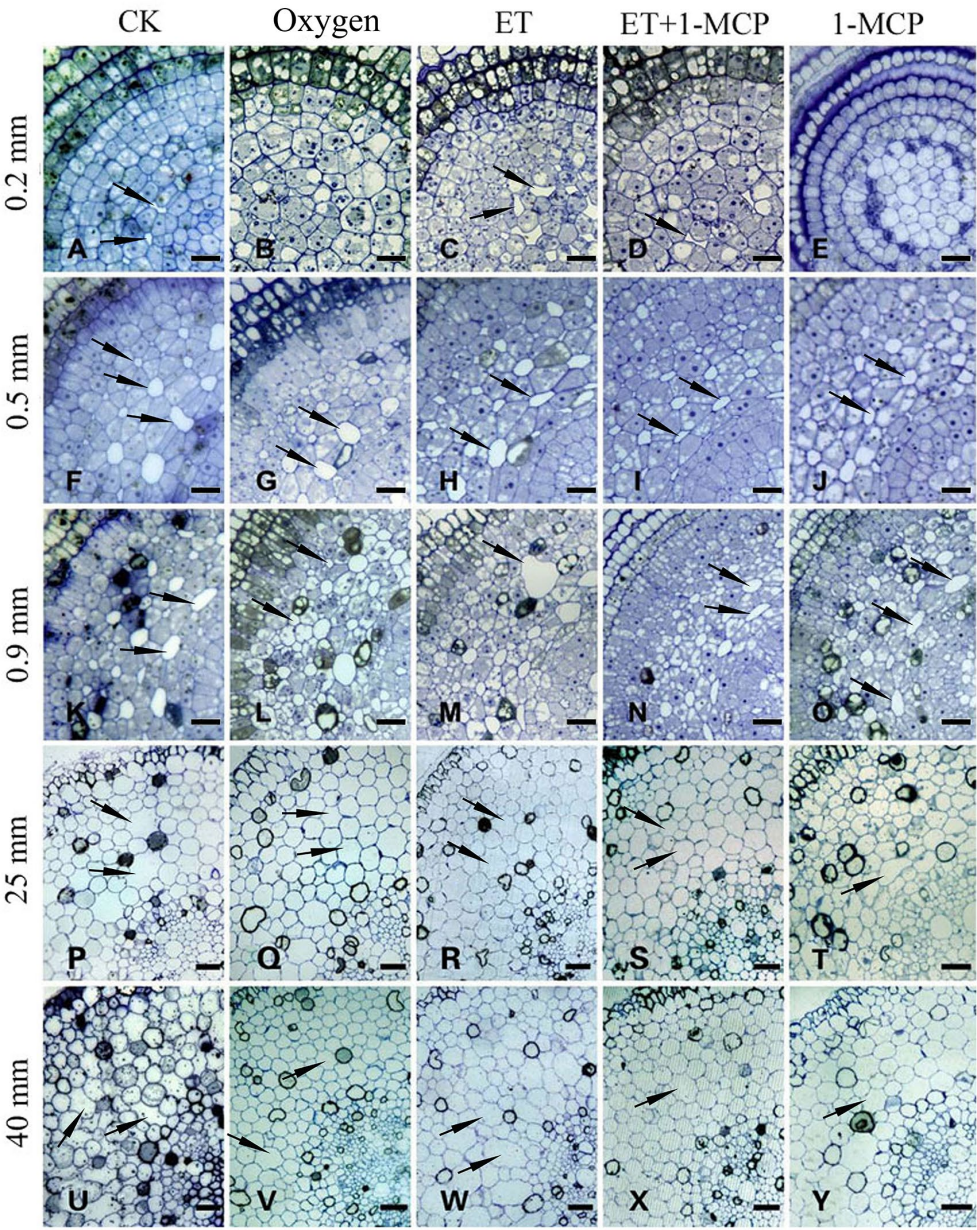
Oxygen and ET induce the aerenchyma formation in *Nelumbo nucifera* roots. **(A–E)** Transverse section of aerenchyma at 0.2 mm from the root tip. **(A)** Control (normal growth); **(B)** 21% oxygen treatment; **(C)** 150 μm ET treatment; **(D)** 150 μm ET + 5 mg/L 1-MCP treatment; **(E)** 5 mg/L 1-MCP treatment. **(F–J)** Transverse section of aerenchyma at 0.5 mm from the root tip. **(F)** Control (normal growth); **(G)** 21% oxygen treatment; **(H)** 150 μm ET treatment; **(I)** 150 μm ET + 5 mg/L 1-MCP treatment; **(J)** 5 mg/L 1-MCP treatment. **(K–O)** Transverse section of aerenchyma at 0.9 mm from the root tip. **(K)** Control (normal growth); **(L)** 21% oxygen treatment; **(M)** 150 μm ET treatment; **(N)** 150 μm ET + 5 mg/L 1-MCP treatment; **(O)** 5 mg/L 1-MCP treatment. **(P–T)** Transverse section of aerenchyma at 25 mm from the root tip. **(P)** Control (normal growth); **(Q)** 21% oxygen treatment; **(R)** 150 μm ET treatment, **(S)** 150 μm ET + 5 mg/L 1-MCP treatment; **(T)** 5 mg/L 1-MCP treatment. **(U–Y)** Transverse section of aerenchyma at 40 mm from the root tip. **(U)** Control (normal growth); **(V)** 21% oxygen treatment; **(W)** 150 μm ET treatment; **(X)** 150 μm ET + 5 mg/L 1-MCP treatment; **(Y)** 5 mg/L 1-MCP treatment. Bars: **A,B,** **D–J** = 30 μm; **C,** **K–O,** **Q,T,V,W** = 50 μm; others = 60 μm.

At 0.2 mm, CK formed two circles of aerenchyma, and there were 10 cavities in the first circle, whereas 21% oxygen treatment group formed one round of aerenchyma and only four cavities in the first round (Tables 1, 2). The area of the first round aerenchyma was significantly lower than that of the CK group (*p < 0.05*; Figure 8). Moreover, the cortical cells of CK and 21% oxygen treatment groups had a larger volume, regular shape, and dense cytoplasm. Due to the separation between cells, small intercellular spaces began to appear between the 1–3 layers of cells close to the procambium in the CK group; yet, this space was less evident in 21% oxygen treatment groups vs. CK group (Figures 9A,B).

**Table 1:** Ring number on the transverse section of aerenchyma in *N. nucifera* roots.

	0.2 mm	0.5 mm	0.9 mm	25 mm	40 mm
CK	2	3	4	4	4
Oxgyen	1	3	4	4	4
ET	2	4	4	4	4
ET + 1-MCP	1	4	4	4	4
1-MCP	0	3	3	4	4

*Nelumbo nucifera* roots at 0.2, 0.5, 0.9, 25, and 40 mm from the root tip were selected, representing the early formation stage, middle formation stage, late formation stage, expansion stage, and mature stage of aerenchyma, respectively. With a normal water environment as the control, aerenchyma development after continuous filling of 21% oxygen, adding ET and its inhibitor 1-MCP in an aquatic environment was observed, and ring number on the transverse section of aerenchyma in *N. nucifera* roots was counted, Values are mean ± SE (*n* = 6).

**Table 2:** Number of cavities in the first circle aerenchyma of *Nelumbo nucifera* roots.

	0.2 mm	0.5 mm	0.9 mm	25 mm	40 mm
CK	10 ± 1.020	17 ± 1.720	19 ± 2.898	18 ± 2.561	24 ± 1.855
Oxgyen	4 ± 0.748	17 ± 1.673	18 ± 1.600	20 ± 2.608	24 ± 2.098
ET	9 ± 1.625	16 ± 1.960	17 ± 1.020	18 ± 2.098	20 ± 2.449
ET + 1-MCP	4 ± 0.632	16 ± 2.280	15 ± 2.280	15 ± 2.530	22 ± 2.828
1-MCP	0 ± 0	14 ± 2.28	14 ± 2.82	16 ± 1.939	21 ± 2.898

*Nelumbo nucifera* roots at 0.2, 0.5, 0.9, 25, and 40 mm from root tip were selected, representing the early formation stage, middle formation stage, late formation stage, expansion stage, and mature stage of aerenchyma, respectively. With normal water environment as the control, aerenchyma development after continuous filling of 21% oxygen, adding ET and its inhibitor 1-MCP in aquatic environment was observed, and number of cavities in the first circle aerenchyma of *N. nucifera* roots was counted, Values are mean ± SE (*n* = 6).

At 0.5 mm, both CK and 21% oxygen treatment groups formed three circles of aerenchyma, and both had 17 cavities in the first circle (Tables 1, 2), but the area of the first round of aerenchyma in the 21% oxygen treatment group was significantly lower than that in the CK group ( *p* < 0.05; Figure 8). Also, the cortical cells in the CK and 21% oxygen treatment groups had a larger volume, regular shape, and dense cytoplasm, and small intercellular spaces began to appear between the three and four layers of cells near the procambium due to the separation of cells, and the number and volume of aerenchyma were increased compared to 0.2 mm stage (Figures 9F,G).

At 0.9 mm, CK formed four-circle aerenchyma, and there were 19 cavities in the first circle; the 21% oxygen treatment group formed four-round aerenchyma, and there were 18 cavities in the first round (Tables 1, 2). The area of the first circle aerenchyma was lower in the 21% oxygen treatment group vs. the CK group, yet, without statistical significance (Figure 8). The cells containing osmiophilic granules could be observed in the CK and 21% oxygen treatment groups (Figures 9K,L).

At 25 mm, CK formed four-circle aerenchyma and 18 cavities in the first round, 21% oxygen treatment group formed four-circle aerenchyma and 20 cavities in the first round (Tables 1, 2). There was no significant difference in the area of the first round aerenchyma compared with CK (Figure 8). A few cells containing osmiophilic granules were still observed in the cortex of the CK and 21% oxygen treatment groups (Figures 9P,Q). At 40 mm from the root tip, both CK and 21% oxygen treatment groups formed four rounds of aerenchyma, and both 24 cavities in the first round (Tables 1, 2). Again, there was no significant difference in the area of the first round aerenchyma compared with CK (Figure 8). A few cells containing osmiophilic granules were still observed in the cortex of the CK and 21% oxygen treatment groups (Figures 9U,V).

### The effect of ET and its inhibitor on the formation of aerenchyma

*Nelumbo nucifera* roots at 0.2, 0.5, 0.9, 25, and 40 mm from the root tip were selected, representing the early formation stage, middle formation stage, late formation stage, expansion stage, and mature stage of aerenchyma, respectively. The development of aerenchyma after adding ET and its inhibitor 1-MCP in the aquatic environment was observed.

At 0.2 mm, CK formed two circles of aerenchyma, and there were 10 cavities in the first circle; the ET treatment group formed two circles of aerenchyma, and there were nine cavities in the first circle; the area of the first circle of aerenchyma was significantly increased compared with CK (*p < 0.05*). ET + 1-MCP treatment group formed one circle of aerenchyma with four cavities; no significant difference was observed in the area of the first circle of aerenchyma compared with CK. No aerenchyma was seen in the 1-MCP treated group (Figure 8; Tables 1, 2). Also, the intercellular spaces appeared between the 1–3 layers of cells near the procambium in the CK and ET treatment group, and the area of the intercellular spaces in the ET treatment group was increased compared with CK. In the ET + 1-MCP treatment group, a few intercellular spaces appeared between the 1–2 layers of cells near the procambium, and no obvious intercellular spaces were observed in the 1-MCP treatment group (Figures 9A,C–E).

At 0.5 mm, the ET treatment group formed four circles of aerenchyma and had 16 cavities in the first circle, and there was no significant difference in the area of the first circle aerenchyma compared with the CK group. In the ET+ 1-MCP treatment group, four circles of aerenchyma were formed, and there were 16 cavities in the first circle; the area was significantly lower than that of CK (*p < 0.05*). The 1-MCP treatment group formed three circles of aerenchyma, with only 14 cavities in the first circle, and its area was significantly lower than that of CK (*p < 0.05*; Figure 8; Tables 1, 2). In the ET treatment group and the ET + 1-MCP treatment group, intercellular spaces were observed in the 1–5 layers of cells near the procambium due to the separation between cells, whereas in the CK and 1-MCP treatment groups, obvious intercellular spaces were observed only in the 1–4 layers cells near the procambium (Figures 9F,H–J).

At 0.9 mm, four circles of aerenchyma were formed in both ET and ET + 1-MCP treatment groups, and 15–17 cavities in the first circle. There was no significant difference in the area of the first circle aerenchyma between the two groups compared with CK. In the 1-MCP treatment group, only three circles of aerenchyma were formed, and 14 cavities in the first circle. The area of the first circle aerenchyma was significantly lower than that of CK (*p < 0.05*; Figure 8; Tables 1, 2). In the ET, ET + 1-MCP, and 1-MCP treatment groups, more aerenchyma, and cells containing osmiophilic granules were observed in the cortex, but small cavities in the ET treatment group fused and formed larger ones (Figures 9K,M–O).

At 25 mm away, the ET and ET + 1-MCP treatment groups formed four circles of aerenchyma and 15–18 cavities in the first circle. The area of the first circle aerenchyma in the two groups was significantly increased compared with that in the CK (*p < 0.05*). In the 1-MCP treatment group, three circles of aerenchyma were formed, and 16 cavities in the first circle. The area of the first circle aerenchyma had no significant difference compared with that of CK (Figure 8; Tables 1, 2). In the ET and the ET + 1-MCP treatment groups, some cells containing osmiophilic granules were observed in the cortex and even in the vascular cylinder, whereas in the 1-MCP treatment group, it was only observed in the cortex (Figures 9P,R–T).

At 40 mm, CK, ET, ET + 1-MCP, and 1-MCP treatment groups all formed four circles of aerenchyma and 20–22 cavities in the first circle. The area of the ET treatment group was significantly increased compared with CK (*p < 0.05*). In ET + 1-MCP, the 1-MCP treatment group, there was no significant difference in the area of first circle aerenchyma compared with CK (Figure 8; Tables 1, 2). In the ET and ET + 1-MCP treatment groups, some cells containing osmiophilic granules were observed in the cortex and vascular cylinder, whereas only a small amount was observed in the 1-MCP treatment group (Figures 9U,W–Y).

## Discussion

### PCD is involved in the formation of aerenchyma in *Nelumbo nucifera* root

In this study, aerenchyma development in *N. nucifera* root was observed. Aerenchyma was mainly caused by increased cell volume and the separation between cells in the formation stage (Figures 1E–G). Subsequently, the cells surrounding the intercellular space gradually shrank, and some special cells were degraded to form cavities in the cortex. Some cells’ contraction, deformation, and final degradation were intuitively observed from the microstructure (Figures 1I,J), thus forming some radially prolonged aerenchyma cavities (Figures 1K,M). Then, the ultrastructure of PCD and the degradation of nuclear DNA were further studied to supplement the series of cytological events during aerenchyma formation. Nuclear DNA fragmentation during aerenchyma formation in *N. nucifera* root can be detected by TUNEL assay (Figure 2). The processes of vacuole membrane rupture, vesicle appearance, organelle degradation, nuclear chromatin condensation and marginalization, plasma membrane rupture, and cell wall degradation were observed in *N. nucifera* root (Figures 4–7). Gunawardena et al. (2001) observed similar cytological events during the aerenchyma formation in maize roots. This evidence confirmed that aerenchyma formation is accompanied by cell death from overall cell level, subcellular level and molecular biology level. There was no diffuse tailing phenomenon in DNA gel electrophoresis (Figure 3), which might be due to plant species specificity.

Jung et al. (2008) compared and analyzed the structures of mature tissues in 110 wetland plants, and the aerenchyma in *Typha angustifolia* leaves were clearly divided into schizogenous types. Furthermore, Ni et al. (2014) further proved the formation of lysogenic aerenchyma in *Typha angustifolia* by studying the morphogenesis and ultrastructure of aerenchyma and cell death detection technology of nuclear fragmentation. In addition, Gunawardena et al. (2004, 2005) reported that the leaves of lace plants and *Monstera obliqua* (Araceae) from perforations through the death of some cells; yet, no cell dissolution were seen in the mature structure. Therefore, the type of aerenchyma cannot be judged only from the histology of mature aerenchyma.it is necessary to continuously observe the occurrence and development of aerenchyma and combine with the cytology technology of cell apoptosis detection, scientifically and accurately classify the aerenchyma.

In addition, osmiophilic granules were also observed in some cells during the aerenchyma development, which seems to be caused by the gradual degradation of the thylakoid membrane in plants under aging or stress (Papadakis et al., 2004), a typical feature of chloroplast aging (Spundova et al., 2003). Previous studies have also found that the size and number of osmiophilic granules in chloroplasts increase dramatically with premature senescence in *Nicotiana tabacum* L (Zhao et al., 2018). Moreover, a large increase in the number of osmiophilic granules was found in chloroplasts of aging *Ginkgo* leaves (Wei et al., 2013). In this study, some cells containing osmiophilic granules were observed in the cortex of *N. nucifera* root, indicating that hypoxia stress caused oxidative stress in some specific cortical cells, resulting in the destruction of the membrane system and the production of many lipid substances. It is speculated that these cells may gradually degrade and eventually die to form the cavity characteristic for aerenchyma. In summary, the expansion of cortical cell volume and the separation between cells form small intercellular spaces in the early stage of aerenchyma formation in *N. nucifera* root, followed by specific cells degrade and clear up *via* PCD, case by oxidative stress under a hypoxic environment, and finally forming aerenchyma. Similarly, during the aerenchyma formation in *T. aestivum*, schizogenesis occurs in the early stage and lysogenesis occurs in the later stage (Song et al., 2009). The aerenchyma in *T. pseudoincisa* and *C. dactylon* were also reported as schizo-lysigenous aerenchyma (Ni et al., 2018; Yuan et al., 2022). The results of this experiment were consistent with those of previous studies. Therefore, aerenchyma in *N. nucifera* root belongs to the schizo-lysigenous.

### Cytological events of PCD

Campbell and Drew (1983) studied aerenchyma formation in maize seedlings root under hypoxia and found that plasma membrane invagination is the first sign that appears. We also found that plasma membrane invagination occurred in the early stage of PCD (Figure 4A), and the cytoplasm began to gradually degrade (Figures 4F,G). The rupture of the vacuole membrane is an important event in the PCD process, and it is also the substantive stage of PCD (Obara et al., 2001). Hydrolases that can degrade cell components are gathered in the vacuole. The rupture of the vacuole membrane during the conduits differentiation in *Zinnia* is an early event of PCD (Fukuda, 1996, 2000; Groover et al., 1997). In this experiment, local vacuole membrane degradation and membrane-like structures were observed in the early stage of PCD (Figure 4H). Key features of PCD, such as nuclear membrane collapse, chromatin condensation, and marginalization, were also observed in the early stage (Figure 5C). In addition, another key feature of PCD, DNA-specific cleavage, was also detected in the early stage of PCD by the TUNEL assay (Figure 2). It is speculated that the release of nuclease after the rupture of the vacuole membrane may lead to nuclear degradation (Fukuda, 2000; Gunawardena et al., 2004). But this is not the only factor; the activation of related enzymes such as caspase-like protein may also lead to this phenomenon.

In the middle stage of aerenchyma development, the cytoplasmic components of the cortical parenchyma cells were squeezed at the edge of the cell by the large central vacuole, which made the cytoplasm condensed and the electron density increased again (Figures 5D–F). At the same time, the plasma membrane was observed to fold inward (Figure 5G). It was also found that the cytoplasmic vacuolization resulted in condensed cytoplasm during aerenchyma formation in *Sagittaria lancifolia* root (Schussler and Longstreth, 2000). Afterward, the cytoplasm was degraded again as PCD continued, and many vesicles appeared in the cytoplasm (Figure 5H). These vesicles were transported to the outside of the plasma membrane by exocytosis, and some vesicles contained black particles (Figures 6A–D). At the same time, it was also observed that organelles began to degrade, showing the disorder of the membrane system and the distortion of organelles morphology. Also, single-layer membrane organelles degraded earlier, and double-layer membrane organelles degraded later (Figures 6E,F), which was similar to the results of previous studies (Fukuda, 1996, 2000; Groover et al., 1997; Gunawardena et al., 2004).

At the late stage of aerenchyma development, the plasma membrane ruptured, and the protoplasts rapidly degraded after losing the protection of the plasma membrane (Figures 7A,B). The degradation of the cell wall was the last step of PCD. Under the electron microscope, the cell wall gradually became thinner and was completely degraded with time (Figure 7D). Under the anoxic condition, the cell wall of maize seedlings changes from electron opacity to electron transparency, and this change appears in the late stage of PCD (Campbell and Drew, 1983). Gunawardena et al. (2001) labeled the esterified and non-esterified pectin with antibodies and found that the cell wall had changed at the early stage of PCD. However, the degradation of the cell wall was not observed until the late stage under the electron microscope, indicating that when no changes in cell wall were observed under the electron microscope, the cell wall had changed as an early event of cell death, so the degradation of cell wall needs further study. According to the cytological characteristics of PCD during aerenchyma formation in *N. nucifera* root, we found that vacuoles had a key role in the PCD events.

### Hypoxic stress is an important factor in the formation of aerenchyma in *Nelumbo nucifera* root

Some studies have suggested that gradually exposing plants to a hypoxic environment is conducive to the formation of aerenchyma and thus improves the anti-hypoxia ability of plants (Dolferus et al., 1997; Germain et al., 1997; Ellis and Setter, 1999). However, in the case of sudden hypoxia, the root tip cells of plants die because their adaptation to stress is too short (Roberts et al., 1984; Johnson et al., 1989; Subbaiah and Sachs, 2003). To adapt to the water environment or wet soil environment, plants have evolved some strategies in the long-term evolution. For example, the roots of some wetland plants form constitutive aerenchyma, and the development degree of aerenchyma increase with the decrease of oxygen content in the growth environment (Colmer et al., 2006; Shiono et al., 2011). Arid plant wheat does not form aerenchyma in well-drained soil; yet, when exposed to hypoxia, lysigenous aerenchyma can appear (Arikado and Adachi, 1955; Drew et al., 1981; Haque et al., 2010). Gunawardena et al. (2001) exposed the roots of maize seedlings to 21% oxygen environment for 2.5 days and found no aerenchyma. However, maize seedlings grown in 3% oxygen environment for 0.5 days formed obvious aerenchyma in the central cortex of roots. In this study, we found that *N. nucifera* root could form aerenchyma under hypoxia stress and sufficient oxygen, indicating that the formation of aerenchyma in *N. nucifera* root was inherited. In addition, we also found aerenchyma in 0.2 mm *N. nucifera* root grown in a normal water environment. However, exposing samples to 21% oxygen in the rhizosphere environment of *N. nucifera*, resulted in absences of aerenchyma at 0.2 mm from the root tip, and the area of aerenchyma in root hair area was smaller than that of the control (Figure 9). Therefore, the formation of aerenchyma is an effective mechanism for reducing hypoxia stress. Hypoxia can induce the occurrence of aerenchyma in plants. Yet, under sufficient oxygen, the formation of aerenchyma is inhibited to a certain extent. The aerenchyma in *N. nucifera* root was constitutive, and hypoxia stress could induce the further development of the aerenchyma in *N. nucifera* root, which is consistent with previous studies (Gong et al., 2019).

### ET induces the formation of aerenchyma in *Nelumbo nucifera* root under hypoxia stress

Ethylene as a hormonal signal regulates the formation of aerenchyma in many plants, such as maize (Gunawardena et al., 2001), wheat (Watkin et al., 1998), pea (Gladish and Niki, 2008), and rice (Justin and Armstrong, 1987; Steffens et al., 2011). Studies have found that ET synthesis in plants increases under hypoxic conditions (Lorbiecke and Sauter, 1999). Hypoxia signal induces the expression of ACS and ACO genes in oxygen-tolerant genotype cotton, resulting in more ET production in this genotype cotton (Pan et al., 2022). Steffen et al. treated rice with 150 μM ET and found that the proportion of aerenchyma increased from 64.6 to 89.8% after 2 days and to 100% after 4 days (Steffens et al., 2012). However, the formation of aerenchyma in some plants, such as *Juncus effususus*, is not dependent on ET (Visser and Bögemann, 2006). In our study, ET not only induced the earlier occurrence of aerenchyma in *N. nucifera* root but also the area of aerenchyma became larger than that of the control; 1-MCP, an ET synthesis inhibitor, could inhibit the occurrence of aerenchyma to some extent (Figure 9), thus indicating that ET, as a signal molecule, is directly involved in the formation of aerenchyma in *N. nucifera* root and has a positive regulatory effect.

## Conclusion

This study confirmed the occurrence of PCD during the development of aerenchyma in *N. nucifera* root by histology, cytology, and molecular biological methods, the type of aerenchyma in *N. nucifera* root is schizo-lysigenous. According to the cytological characteristics of PCD during the formation of aerenchyma in *N. nucifera* root, we found that vacuoles have a key role in PCD events associated with the formation of lysogenic aerenchyma in *N. nucifera* root. Moreover, the aerenchyma in *N. nucifera* root was formed by the active death of some cortical cells during differentiation, which was used for gas exchange *in vivo*, so its PCD events also belong to the differentiation and development type.

Aerenchyma began to form at 0.2 mm from the root tip in the normal aquatic environment. No aerenchyma was formed at 0.2 mm from the root tip after continuous filling with 21% oxygen in the rhizosphere environment of *N. nucifera*, and the area of aerenchyma in the root hair area was smaller than that in control. These data suggest that hypoxia stress is an important factor in forming aerenchyma in *N. nucifera* root. On the contrary, sufficient oxygen can inhibit the formation of aerenchyma to a certain extent. At the same time, we found that *N. nucifera* root could form aerenchyma under hypoxia stress and sufficient oxygen; its aerenchyma was controlled genetically. Therefore, the type of aerenchyma was constitutive, and hypoxia stress could induce the further development of aerenchyma in *N. nucifera* root.

Ethylene could not only induce the earlier occurrence of aerenchyma in *N. nucifera* root under hypoxia stress, but also the area of aerenchyma became larger than that of the control. 1-MCP, an ET synthesis inhibitor, could inhibit the occurrence of aerenchyma to some extent, thus indicating that ET, as a signal molecule, was directly involved in the formation of aerenchyma in *N. nucifera* root under hypoxia stress and had a positive regulatory effect. In subsequent studies, this conclusion can be further proved by sampling and analyzing PCD-related gene expression and signaling pathways at different time points.

## Data availability statement

The original contributions presented in the study are included in the article/supplementary material; further inquiries can be directed to the corresponding author.

## Author contributions

NXL and HH designed the experiments; XQM, HH and YPX performed the experiments; YPX, ZHY and LYZ analyzed the experimental results; XQM wrote the manuscript. LXB, CL, HY, PDB and NXL revised the manuscript. All authors read and approved the final manuscript.

## Funding

This study was financially supported by the Ningxia Key Research and Development Program (2020BFG03006 and 2021BEG02005), National Natural Science Foundation of China (31960038 and 31660045), and the National Natural Science Foundation of Ningxia (2020AAC03107 and 2021AAC03017).

## Conflict of interest

The authors declare that the research was conducted in the absence of any commercial or financial relationships that could be construed as a potential conflict of interest.

## Publisher’s note

All claims expressed in this article are solely those of the authors and do not necessarily represent those of their affiliated organizations, or those of the publisher, the editors and the reviewers. Any product that may be evaluated in this article, or claim that may be made by its manufacturer, is not guaranteed or endorsed by the publisher.

## Abbreviations

CK: Control
PCD: Programmed cell death
ET: Ethylene
1-MCP: 1-methylcyclopropene
C: Cavity
COR: Cortex
CW: Cell wall
E: Epidermis
GT: Ground tissue
M: Mitochondria
N: Nucleus
P: Plastid
PLA: plasma membrane
PT: Protoderm
PC: Procambium
SG: Starch grain
V: Vacuole
VB: Vascular bundle
Ve: Vesicle
TUNEL: Terminal deoxynucleotidyl transferase-mediated dUTP nick end labeling
DAPI: 4′6-diamidino-2-phenylindole
